# Impact of Heartfulness meditation practice on anxiety, perceived stress, well-being, and telomere length

**DOI:** 10.3389/fpsyg.2023.1158760

**Published:** 2023-06-05

**Authors:** Mansee Thakur, Yogesh Patil, Sanjana T. Philip, Tahreem Hamdule, Jayaram Thimmapuram, Nishant Vyas, Kapil Thakur

**Affiliations:** ^1^Department of Medical Biotechnology, Central Research Laboratory, Mahatma Gandhi Mission School of Biomedical Sciences, Mahatma Gandhi Mission Institute of Health Sciences, Navi Mumbai, India; ^2^Department of Internal Medicine, Well Span York Hospital, York, PA, United States; ^3^Logical Life Science, Pvt. Ltd., Pune, India; ^4^SRCM Heartfulness Meditation Centre, Navi Mumbai, India

**Keywords:** anxiety, cortisol, Five Facet Mindfulness Questionnaire (FFMQ), Heartfulness meditation, RT-PCR, stress, telomere

## Abstract

**Objective:**

Exhaustion, stress, and burnout have all been found to be reduced using techniques like yoga and meditation. This study was carried out to check the effectiveness of Heartfulness practice (a form of meditation) on certain psychological and genetic variables.

**Methods:**

A total of 100 healthy individuals (aged 18–24) were recruited and randomized into two groups-Heartfulness intervention and control group. The intervention was carried out for 03 months. Participants from both groups were analysed for their cortisol levels and telomere length before and after the intervention. Psychometric measures of anxiety, perceived stress, well-being and mindfulness were carried out using Beck Anxiety Inventory (BAI), Perceived Stress Scale (PSS), WHO-Well-being Index (WHO-WBI) and Five Facet Mindfulness Questionnaire (FFMQ).

**Results:**

The cortisol levels in the meditators group significantly decreased (*p* < 0.001) after the intervention as compared to the non-meditators group, whereas, the telomere length increased in the mediators group. This increase was not significant (*p* > 0.05). Anxiety and perceived stress also decreased post intervention, and well-being as well as mindfulness increased, as assessed by the questionnaire tools, although the decrease in perceived stress was statistically insignificant (*p* > 0.05). A negative correlation was observed between telomere length and cortisol (stress biomarker), whereas a positive correlation was found between telomere length and well-being.

**Conclusion:**

Our data provide evidence that Heartfulness meditation practice can improve our mental health. Additionally, telomere length is shown to be affected by cortisol levels, and this meditation practice can also help to increase telomere length, and thereby slow down cellular aging. However, future studies with larger sample size are required to confirm our observations.

## Introduction

A growing number of people are suffering from complicated lifestyle disorders such as cardiovascular disease (CVD), infertility, diabetes, depression, and cancer. These conditions require special care and treatment to achieve optimal quality of life. These diseases have become a burden on modern society because they are significantly linked to accelerating cellular aging (Boccardi et al., 2016; Tolahunase et al., 2017). Major depressive disorder (MDD) is associated with a significantly increased risk of developing serious medical illnesses that are more commonly seen with advanced age, such as diabetes, cardiovascular disease, immune impairments, stroke, dementia and osteoporosis. A major depressive episode has been compared to “accelerated ageing,” with an increased risk of aging-related disorders (Thimmapuram et al., 2017). The human body undergoes constant changes, causing changes in cells and tissues over the course of a lifetime. Some changes that occur in cells are normal processes that help keep the body healthy. Other changes can occur as a result of certain diseases or conditions, such as cancer or aging (Lipton, 2008). Certain cells in the body have specialized structures called telomeres, which are the protective caps found at the end of chromosomes that shorten with each cell division. Eventually, if the telomeres are too short, the cells cannot divide anymore and become damaged, which can lead to cell death. Cellular senescence is another consequence of shortened telomeres (Blackburn, 2000). Telomere length has an impact on total life expectancy, and telomere shortening is a sign of molecular aging (Blackburn, 2009; Beery et al., 2012; Karthik et al., 2014; Alda et al., 2016; Thimmapuram et al., 2017). Telomere shortening has been linked to cytotoxic stresses such as oxidative stress, which destroys telomeric DNA more than non-telomeric DNA, and chronic inflammation, even in non-dividing cells. Increased telomere shortening makes cells more vulnerable to apoptosis and death (Wolkowitz et al., 2011).

Cortisol is a stress hormone that is produced by the adrenal glands. Research has shown that under normal circumstances, the body maintains or regulates normal cortisol levels. However, if under higher stress conditions, the body secretes more of this hormone (Lengacher et al., 2014). Cortisol is also responsible for several stress-related changes in the body. The concentration of cortisol has also been reported to be a useful prognostic marker of stress (Álvarez-López et al., 2022).

Exhaustion, stress, weariness, and burnout have all been found to be reduced using techniques like yoga and meditation (Walsh and Shapiro, 2006; Thimmapuram et al., 2017). Yoga is also effective in treating depression (Cramer et al., 2013; Seppälä et al., 2014; De Manincor et al., 2016; Falsafi, 2016; Cramer et al., 2017; Prathikanti et al., 2017; Ramanathan et al., 2017; Streeter et al., 2017), even in the perinatal period (Cramer et al., 2013; Davis et al., 2015). The results with yoga vs. exercise and yoga vs. medication were found to be similar, as concluded by a systemic review (Cramer et al., 2017). However, results indicating yoga for the treatment of anxiety is unclear. Few studies have found that yoga is effective as compared to no treatment (Michalsen et al., 2012; Parthasarathy and Jaiganesh, 2014; Vorkapic and Rangé, 2014; Falsafi, 2016; Ramanathan et al., 2017), whereas other studies suggest no improvement in anxiety (Davis et al., 2015; De Manincor et al., 2016). Tai chi is another practice that has shown to reduce anxiety in older adults, when used as an adjunct therapy along with medications (Song et al., 2014). A small body of literature indicates mixed evidences for qi gong therapy. A qi-gong-based stress reduction program showed reduced anxiety (Hwang et al., 2013), however a meta-analysis revealed contradictory results (Wang et al., 2010). Tai chi and qi gong therapies have also shown evidence to reduce depression (Tsang et al., 2013; Yin and Dishman, 2014; Yeung et al., 2017). Apart from these strategies, meditation can also be used as an effective treatment strategy for psychiatric disorders such as depression and anxiety. For instance, Mindfulness-Based Interventions (MBIs) showed better results for reducing depression as compared to no treatment. It is also worth noting that this intervention was found to be equivalent to treatment by selective serotonin reuptake inhibitors (Goldberg et al., 2018). Anxiety and mood disorders can also be reduced using MBIs as determined by a meta-analysis (Hofmann et al., 2010). A study showed that depression in patients with a traumatic brain injury was reduced by MBI (Bédard et al., 2014). Similarly, MBI was effective in patients with PTSD and depression, although statistically significant results were not obtained for anxiety and quality of life (Hilton et al., 2017).

In recent years, there has been a growing interest in exploring the possible impacts of meditation practice on telomere dynamics, in addition to diet and physical activity (Dyrbye et al., 2008). In leukocytes, psychological stress has been linked to rapid telomere shortening, whereas meditation has been linked to increased telomere length (Blackburn, 2009; Beery et al., 2012; Karthik et al., 2014; Thimmapuram et al., 2017). Shorter telomeres are also linked with diseased conditions diseases (Zhao et al., 2013; Haycock et al., 2014; Ridout et al., 2016). A growing body of research suggests that meditation, which has been shown to support healthy biological processes, may also affect biomarkers associated with aging. The findings suggest that practicing mindful meditation may be one way to build resilience against disease through maintaining a healthy body and mind (Sudsuang et al., 1991; Pace et al., 2009; Lengacher et al., 2014; Turan et al., 2015; Alda et al., 2016; Álvarez-López et al., 2022). In fact, intensive meditation training has been linked to increased telomerase activity (Jacobs et al., 2011) and longer telomere length in blood cells, which are considered potential biomarkers of human ageing. Recent research suggests that these factors may be influenced by psychological stress, stress assessments, and well-being (Epel et al., 2004; Walvekar et al., 2015; Alda et al., 2016). Investigations have demonstrated a beneficial correlation between meditation and longer telomeres (Alda et al., 2016) in addition to an increase in telomerase (Zi and Shuai, 2013), suggesting that meditation may be crucial for disease prevention.

Heartfulness meditation is one such practice of meditation. This tradition uses three primary methods: 1) meditation, 2) cleaning, and 3) prayer. These techniques are intended to purify and broaden consciousness and awareness about oneself (Sylapan et al., 2020). Heartfulness meditation was chosen because of its characteristic yogic transmission which helps to achieve a state of Samadhi even in beginners due to the effectiveness of Pranahuti. Furthermore, the Heartfulness technique draws upon the research and practical experience of yogis, as opposed to abstract theory. They emphasize “direct perception” as the preferred method, which is considered to be a more accurate method of learning from Yoga (van’t Westeinde and Patel, 2022).

Heartfulness meditation has been shown to positively influence physical and mental health outcomes, however there are relatively few studies exploring its biological mechanisms.

This study was carried out to support the idea that Heartfulness meditation is linked to longer telomeres. The second objective was to link it with decreased cortisol levels and psychological variables such as stress, anxiety, mindfulness and well-being. Only a few researchers have shown this connection (Epel et al., 2004; Zi and Shuai, 2013; Walvekar et al., 2015; Alda et al., 2016). Questionnaire tools such as PSS, BAI, WHO-WBI, and FFMQ were used to show the effect of meditation on different psychological constructs.

## Methodology

### Participants and setting

The study was a prospective cohort analysis carried out for 12 weeks from October 2022–December 2022. It is a single-arm randomized-controlled trial with one intervention condition (guided Heartfulness meditation) and one active-control condition (sham meditation). The design employed was a 2 (condition) × 2 (time) parallel-group design which is explanatory in nature. A convenience sampling method was chosen to recruit healthy-matched (*N* = 100) participants. The principal investigator (PI) sent emails to volunteers to assess their interest in participation. Participants were assigned randomly into two groups-the meditators (intervention) and the non-meditators (no intervention). Participants enrolled had a similar lifestyle and were matched by gender and age (±2 years). Participants in both groups were aged between 18 to 24 years. The participants did not have any experience in any of the meditation practices. According to G Power Software, a sample size of 100 participants (50 meditators and 50 non-meditators) was required for an effect size of 0.8 and 80% power for Type I error; *α* = 0.05 (Faul et al., 2007). 50 participants were randomly allocated into the meditaors group and 50 into the non-meditators group. There was an exclusion from the study for participants with psychiatric disorders, those undergoing pharmacological or psychological treatment or those with medical conditions that may affect the activity of telomerase (such as cancer, lupus, rheumatoid arthritis) (Thimmapuram et al., 2017). To ensure the integrity of the study, all the selected participants were given an information sheet explaining the details about the purpose of the study, voluntary participation, duration of the study, participants’ responsibilities and potential benefits of the study. Written consent was also obtained from each participant. Ethics approval for this study was granted by the Ethics Review Committee, of MGMIHS (MGM/DCH/IEC/109/22).

### Intervention

A Heartfulness-certified trainer briefed the participants of the meditators group on how to practice Heartfulness meditation. These participants practiced the meditation technique once daily on all working days (online-through HeartssApp, and offline) and on their own on holidays by using Heart App software. They meditated weekly once in the Heartfulness meditation centre-located at New Panvel, Navi Mumbai, India.

Guided audio clips were also shared with the participants to follow Heartfulness core practices (meditation, rejuvenation, and bed-time relaxation and meditation) every day for 12 weeks in the following schedule:

**Table:** 

Sr. no.	Practice	Description of practice	Duration (mins)
1.	Heartfulness meditation-morning	Participants were asked to sit comfortably and with their eyes closed, were made to focus their attention on the source of light that is present within the heart. Rather than strictly trying to visualize this, participants were asked to simply tune in to their hearts and be open to any experience that they may feel (van’t Westeinde and Patel, 2022)	30
2.	Heartfulness rejuvenation/cleaning-evening	Participants were asked to imagine that stress and heaviness (‘impurities and complexities’) were escaping through the back of their body in the form of smoke or vapour. And, feelings of purity, lightness, and freshness replaced these impurities (van’t Westeinde and Patel, 2022)	15
3.	Heartfulness bed-time relaxation and meditation before sleeping – night time	Participants were asked to recite the Heartfulness prayer, followed by 10 min of meditation in order to strengthen one’s connection to the source (van’t Westeinde and Patel, 2022).	15

The non-meditators group did not receive any intervention of Heartfulness meditation program during the study period. The group was requested to complete the same baseline assessments as the intervention group and were instructed to carry on with their usual daily life routine.

Participants of both groups had to go through tests of selected psychological, biological and molecular parameters before and after 3 months (ie. Pre-test and Post-test).

#### Procedure

Participants from both groups gave their blood samples in the morning (fasting) for measurement of telomere length and cortisol levels. They were also asked to complete sociodemographic, psychological, and health-related questionnaires. All these analyses/measurements were carried out at MGM Central Research Laboratory, MGMIHS, Navi Mumbai.

### Measurements

#### Telomere length measurement by using qRT-PCR

The telomere length for pre and post intervention was analysed as per the protocol described by Cawthon (2002). A total of 80 samples were analysed by qRT-PCR assay. All extracted DNA samples were normalized to final concentration of 10 ng/μL. The telomere length and housekeeping gene (acidic ribosomal phosphoprotein 36B4) specific PCR was performed by using Takara’s TBGreen^®^ Premix Ex Taq^™^ II PCR master mix by mixing 10 μL of TB Green Premix Ex Taq II (TliRNaseH Plus) (2X), 0.8 μL forward and reverse primers with a final concentration of 10 μM, 0.4 μL ROX Reference Dye (50X), 6 μL of molecular grade water and 2 μL template DNA samples (10 ng/μL). The prepared samples were subjected to following thermal cycling conditions, 95^0^C for 5 min as an initial denaturation followed by 45 cycles of 95^0^\u00B0C for 5 s and 60^0^\u00B0C for 30 s. Post amplification the average CT values were calculated for all analysed samples and the T/S ratio was calculated by using the method described by Cawthon (2002). TL is expressed as t/s, the ratio of telomeric (T) to single copy (S) gene product for a particular sample. T and S values were measured in triplicate using a real-time PCR machine with a 96-tube capacity performed using Himedia Insta Q48 real-time PCR system, and the t/s ratio for a given sample was calculated.

#### Cortisol measurement by competitive ELISA

A total of 80 samples were analysed for quantitative estimation of serum cortisol as an indicator of stress marker by using Bioelsa Competitive ELISA method. The blood samples were collected in the morning. The serum samples were diluted 10 folds in order to get the concentrations of the unknown samples within the detection limits of the said assay, rest of the assay protocol was followed according to the standard operating procedure as prescribed by the manufacturer. The analysis of the results was done by plotting the concentration verses O. D plot of standard reference samples and the concentration of the unknown samples was calculated as per the standard graph.

##### Wellbeing questionnaires

Four self-reporting inventories were used in this study, namely, the Beck Anxiety Inventory (BAI), Perceived Stress Scale (PSS), WHO Well-being Index (WHO-WBI) and the Five Facet Mindfulness Questionnaire (FFMQ).

##### Beck anxiety inventory (BAI)

To measure anxiety among paramedical students. The BAI questionnaire; a 21-item questionnaire, has been commonly used in clinical research as a measure of generalized anxiety (physical and cognitive anxiety). It is a trademark of Pearson Education, Inc., or its affiliate(s). A four-point Likert scale ie. 0 (not at all) to 3 (severely), is used to score the responses. It consists of items that indicate how much a person has been bothered by that symptom during the past month. For example, a sample item in the scale is: “Fear of worst happening.” A score of 36 and above indicates high anxiety, 22–35 moderate anxiety, and 0–21 low anxiety (Piotrowski, 1999).

##### Perceived stress scale (PSS)

To measure perceived stress among the students, the PSS questionnaire; a 10-item questionnaire tool was used. It evaluates the degree at which each individual perceives situations in their lives as stressful. It is widely used for young people and adults above 12 years. Here, a 5-point Likert scale ie. 0 (never) to 4 (very often) is used. A sample item is: “In the last month, how often have you felt difficulties were piling up so high that you could not overcome them?” High levels of perceived stress is determined by scores in the range from 27 to 40, moderate perceived stress by 14–26 and low perceived stress by 0–13 scores (Lee, 2012).

##### WHO-well being index (WHO-WBI)

It is a short questionnaire to measure well-being over the last 2 weeks. It was used to indicate the well-being of the students. It consists of 5 items, each rated between 0 and 5 representing “At no time” and “All the time” respectively. A sample item is: “I have felt cheerful and in good spirits.” Scores between 0 and 25 indicates the worst and best possible lifestyle (Topp et al., 2015).

##### Five facet mindfulness questionnaire (FFMQ)

It is a self-reported questionnaire to measure the tendency to be mindful in daily life. This questionnaire assessed students’ mindfulness which included various parameters such as observing, describing, acting with awareness, non-judging of inner experience, and non-reactivity to inner experience. A sample item is: “I pay attention to sensations, such as the wind in my hair or sun on my face.” A 5-point Likert scale ie. From 1 (never or very rarely true) to 5 (very often or always true), was used (Baer et al., 2008). All these three questionnaires were sent electronically in the form of Google Forms to all participants pre-and post-intervention, and their responses were collected.

### Statistical analysis

Depending on the variable, mean, standard deviation (SD), or percentage values were used. Mann Whitney U test was performed to calculate the significance of psychological variables between the groups. Student’s *t* test and chi-square test were performed for the sociodemographic characteristics of the samples. Pearson correlation (*r*) was used to study and determine the relationship between the psychological variables, telomere length, and cortisol levels. All these tests were performed at the significance level *α* < 0.05 by the SPSS-25 statistical software package.

## Results

Out of 100 participants enrolled in this study, a total of 18 participants were retained till the end of this study. They were randomly allocated into meditators (intervention) and non-meditators group (no intervention); 40 participants were in each group. The sociodemographic characteristics (age, age groups, and gender) of the sample are tabulated in Table 1. Both the meditators and non-meditators groups were similar in age and gender.

**Table 1:** Sociodemographic characteristics of the sample.

Characteristics	Meditators	Non-meditators	Significance
1. *Age*			*p* = 0.327^a^
Mean (SD)	20.95 (1.753)	21.35 (1.875)	
Range	18–24	18–24	
2. *Age groups-n, (%)*			
<18	0	0	
18 to <20	5 (12.5)	4 (10)	
20 to <22	24 (60)	19 (47.5)	
≥22	11 (27.5)	17 (42.5)	
3. *Gender-n, (%)*			*p* = 0.576^b^
Male	9 (22.5)	7 (17.5)	
Female	31 (77.5)	33 (82.5)	

a *p* values were calculated using the independent *t*-test.

b *p* values were calculated using the Chi-square test.

### Telomere length measurement by using qRT-PCR

**Figure 1:**
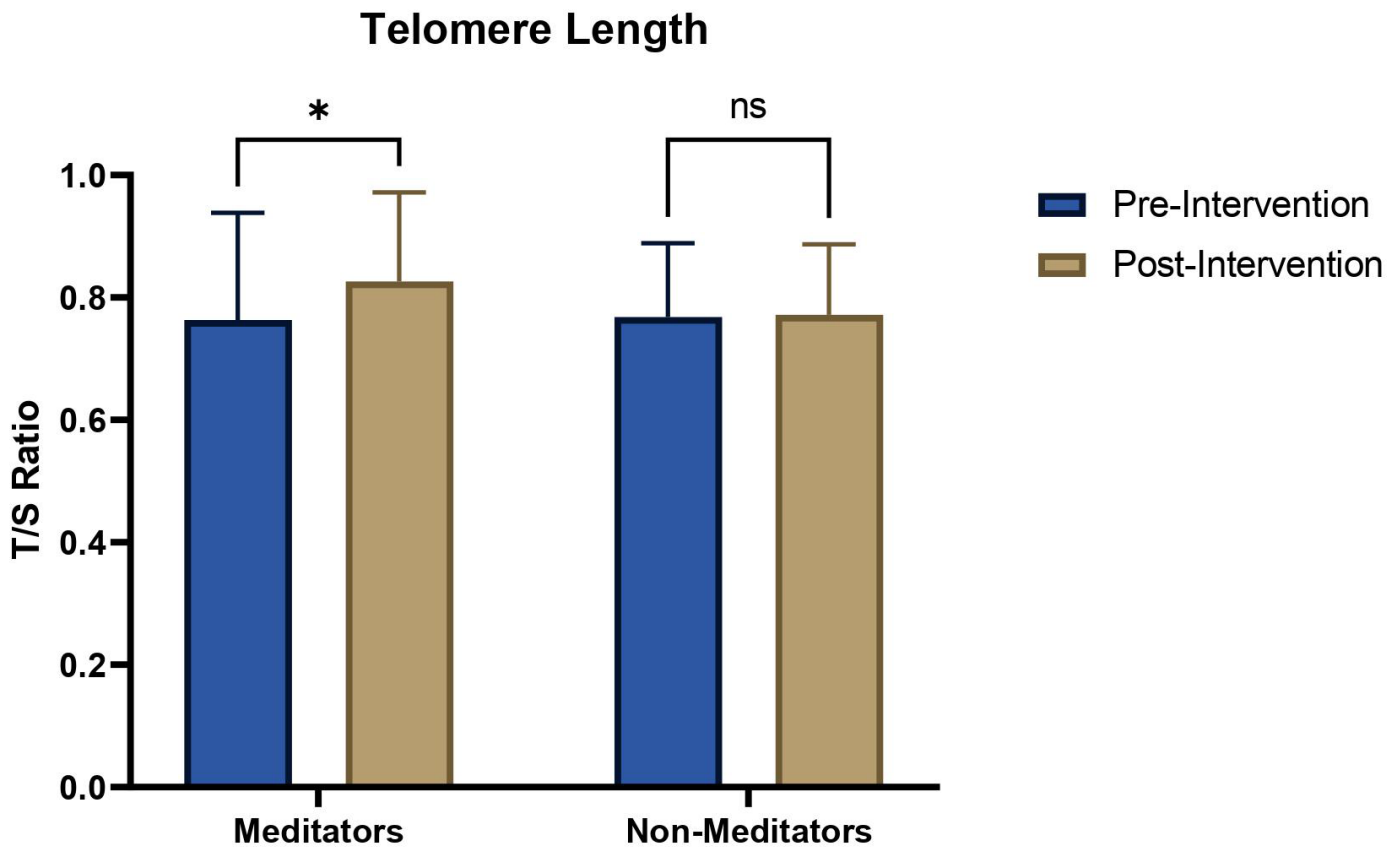
Telomere length in meditators and non-meditators at pre and post-intervention of Heartfulness meditation practice. A significant increase in telomere length was observed in meditators after the intervention period. *p<0.05.

The telomere length was measured using qRT-PCR. The average telomere length in the meditators group (mean = 0.83; SD = 0.144) was found to be more (*t* = 1.656; df = 78; *p* = 0.0190) than the non-meditators group (mean = 0.77; SD = 0.114), as calculated by paired *t*-test (Figure 1).

### Cortisol measurement by competitive ELISA

**Figure 2:**
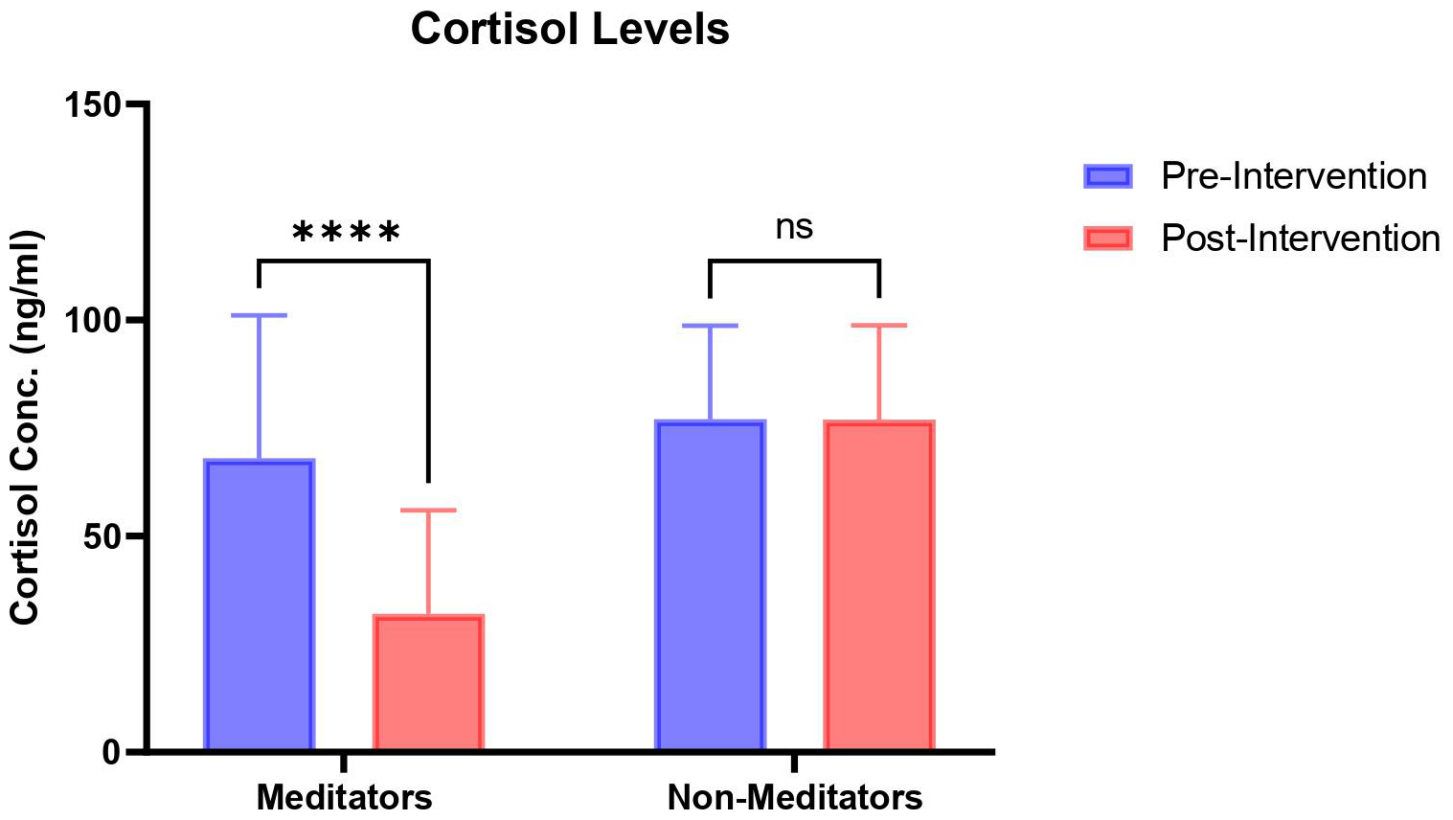
Concentration (ng/mL) of cortisol in meditators and non-meditators at pre and post-intervention of Heartfulness meditation practice. A significant decrease in cortisol was observed in meditators post-intervention. ****p<0.0001.

The paired *t*-test showed that the average cortisol level in the meditators group (mean = 32.07 ng/mL; SD = 23.583) was significantly less (*t* = 3.999; df = 78; *p* = 0.0003) than the non-meditators group (mean = 76.975 ng/mL; SD = 21.567) (Figure 2).

### Psychological variables

The psychological variables such as perceived stress, anxiety, FFMQ, and well-being were studied in meditators and non-meditators before and after the intervention. The level of anxiety and perceived stress was higher in non-meditators, as compared to meditators, after the intervention; however, the difference was significant only in the case of anxiety (*p* = 0.008). The mindfulness variables such as observing, describing, and acting with awareness significantly increased in meditators. However, there was no significant difference observed with non-judging and non-reactivity. The well-being scores also increased significantly (*p* = 0.010) after meditation (Table 2).

**Table 2:** Psychological variables in meditators (*n* = 40) and non-meditators (*n* = 40).

Variable	Meditators, mean (SD)	Non-Meditators, mean (SD)	*z* score	*p* value^a^
Perceived Stress^b^	18.6 (7.489)	21.03 (7.433)	−1.003	0.316
Anxiety^c^	12.4 (9.083)	21.28 (14.793)	−2.673	0.008
FFMQ^d^	122.85 (17.876)	98.35 (31.939)	−3.457	0.001
FFMQ Observing	28.55 (6.872)	19.7 (8.582)	−4.216	0.000
FFMQ Describing	24.78 (4.44)	20.87 (6.541)	−2.455	0.014
FFMQ Acting with awareness	22.78 (4.388)	18.2 (6.513)	−3.629	0.000
FFMQ non-judging	18.47 (4.206)	20.03 (6.956)	−1.240	0.215
FFMQ non-reactivity	18.45 (5.198)	18.35 (6.912)	−0.024	0.981
Well-being^e^	12.3 (6.174)	15.73 (6.333)	−2.578	0.010

a *p* values were calculated using the Mann Whitney U test due to non-normalpaired data.

b As determined by the Perceived Stress Scale (PSS).

c As determined by the Beck Anxiety Inventory (BAI).

d As determined by the Five Facet Mindfulness Questionnaire (FFMQ Total).

e As determined by the WHO-Well Being Index (WHO-WBI).

### Telomeres and psychological variables

The correlation between telomere length and psychological variables was in the expected direction. Post-intervention, a negative correlation between perceived stress, anxiety and mindfulness was observed (Table 3); although this correlation was significant only in the case of perceived stress.

**Table 3:** Correlation between psychological variables and telomere length.

Variable	Telomere Length (r)	*p* value
Perceived stress	−0.315**	0.004
Anxiety	−0.211	0.061
FFMQ	−0.056	0.624
Well-being	0.009	0.937

*r* = Pearson’s Coefficient. **p<0.01.

Similarly, the correlation between cortisol and psychological variables was in the expected direction. With an increase in cortisol levels, an increase in perceived stress, and anxiety was also observed (Table 4); however, this correlation was only significant in the case of anxiety. Whereas, a significant decrease in mindfulness and well-being was observed with an increase in cortisol.

**Table 4:** Correlationp between psychological variables and cortisol.

Variable	Cortisol levels (r)	*p* value^a^
Perceived stress	0.003	0.978
Anxiety	0.237*	0.034
FFMQ	−0.308**	0.005
Well-being	−0.221*	0.048

*r* = Pearson’s Coefficient. *p<0.05; **p<0.01.

**Figure 3:**
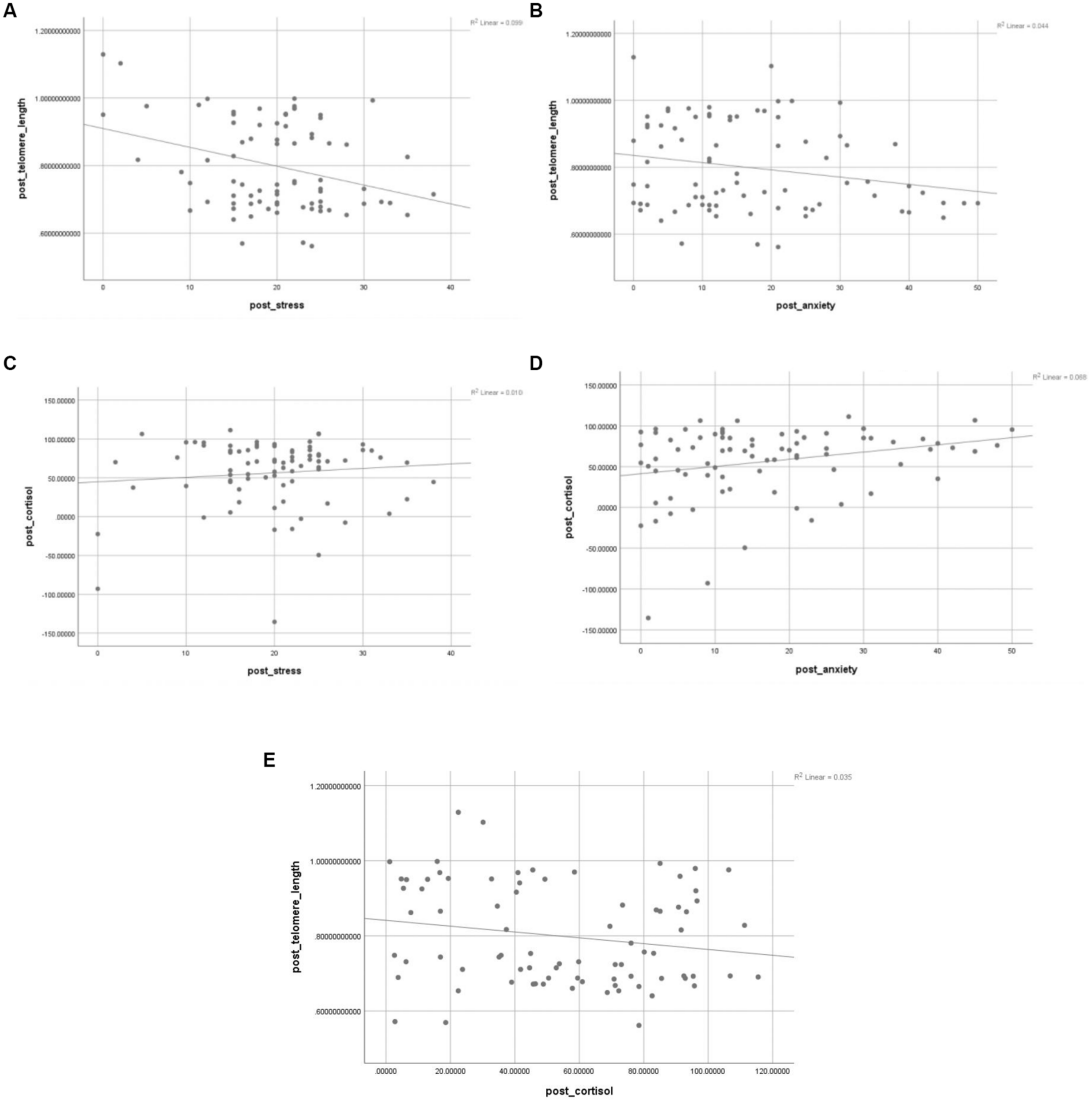
Correlation between psychological variables, telomere length, and cortisol levels. **(A)** correlation between telomere length and stress; **(B)** correlation between telomere length and anxiety; **(C)** correlation between cortisol and stress; **(D)** correlation between cortisol and anxiety; **(E)** correlation between telomere length and cortisol levels.

The scatter plot of psychological variables with cortisol and telomere length is shown in Figure 3.

## Discussion

Our study adds to the growing literature that meditation practice improves mental health. The main hypothesis of our study was that Heartfulness meditation can improve mental health by increasing telomere length. This study demonstrates that Heartfulness meditation affects cortisol levels and psychological variables such as perceived stress, anxiety, mindfulness, and well-being. It further demonstrates that Heartfulness meditation is linked to longer telomeres. All these parameters were studied in two groups; one with the intervention of Heartfulness meditation and the other with no intervention. The non-meditators group did not show any significant differences in any of these parameters. The sociodemographic characteristics of both groups were similar.

It is expected that people with anxiety and stress may have low mindfulness awareness. The same was observed in our study. The PSS and BAI questionnaire revealed that Heartfulness meditation was able to decrease perceived stress and anxiety scores in our participants, although it was not significant in the case of perceived stress. These results are similar to previous studies. For instance, a Mindfulness-based Stress Reduction (MBSR) program in cardiac patients showed statistically significant changes in depression (*p* = 0.01) and anxiety (*p* = 0.04), and a non-significant change in perceived stress (Nijjar et al., 2019). MBSR intervention in students in low-middle-income countries (LMICs) indicated an effective reduction in stress scores. Interestingly, even after 2 months of completion of the intervention, lower negative emotional states, were observed especially in the Anxiety and Depression scores (An et al., 2022). A Mindfulness-based Yoga intervention in nurses and health care professionals (HCPs) showed significant improvement in perceived stress and mindfulness, whereas, cortisol and blood pressure were not significantly improved (Hilcove et al., 2021). Our results reveal that meditators are better at labeling their experiences, such as observing, describing, and acting with awareness which significantly increased after the intervention, suggesting that Heartfulness practice is positively related to mindful awareness. However, there was no significant difference observed with the parameters of non-judging and non-reactivity. The well-being scores also increased significantly (*p* = 0.010) after meditation. These higher mindfulness levels in meditators are consistent with previous work that provides a link between Heartfulness meditation and perceived stress. Thimmapuram et al. (2017) also found that negative emotions, and burnout were reduced with the help of meditation and that further research with more participants and a more representative sample would provide significant results to this effect. The current study indicates that Heartfulness practice can help improve mental well-being and aid in the reduction of anxiety.

The majority of the studies are based on mindfulness practices. To the best of our knowledge, this is the first study to establish a direct effect of Heartfulness meditation practice on the stress hormone cortisol. A significant decrease (*p* = 0.0003) in serum cortisol levels in meditators was observed after the intervention. Previous studies involving other meditation-based interventions have shown similar results. In a study, MBSR intervention in patients with Generalized anxiety disorder (GAD) showed a larger reduction in cortisol as compared to the control group after the Trier Social Stress Test (TSST), showing resilience to stress (Hoge et al., 2018). In another study, a mindfulness retreat showed a statistically significant decrease (*p* < 0.0001) in anxiety and perceived stress (Gardi et al., 2022). This study also showed a positive significant correlation between cortisol levels and both perceived stress (*r* = 0.92, *p* < 0.0001) and anxiety (*r* = 0.56, *p* < 0.0001); as demonstrated in our study too (Gardi et al., 2022). MBSR program has also shown improved effects in lowering cortisol in patients with primary open-angle glaucoma (POAG) (Dada et al., 2018). Other forms of meditation have also shown similar results. For instance, a brief Psychoneuroendocrinoimmunology-Based Meditation (PNEIMED) displayed reduced levels of cortisol levels at awakening and under acute mental stimulation in a group of healthy university students (Bottaccioli et al., 2020). Another group of researchers showed that Rajyoga meditation reduced anxiety and cortisol levels in patients undergoing coronary artery bypass surgery (Kiran et al., 2017).

In the current study, the changes in telomere length were also studied. It was observed that the average telomere length significantly increased (*p* = 0.0190) in the meditators group after the intervention. There are other studies that show longer telomeres (Schutte and Malouff, 2014; Thimmapuram et al., 2017; Tolahunase et al., 2018). Madhuri Tolahunase et al. showed that Yoga and meditation-based lifestyle intervention (YMLI) increased telomerase activity and telomere length, but it was not significant in the latter; whereas a significant decrease in cortisol levels was observed in apparently healthy individuals after YMLI (Tolahunase et al., 2017). A 12-week YMLI showed significantly increased telomerase activity and decreased cortisol levels in patients with Major Depressive Disorder (MDD) (Tolahunase et al., 2018). In a pilot trial involving African American patients suffering from stage 1 hypertension, two interventions-Transcendental Meditation technique plus health education and extensive health education, respectively, showed an increase in gene expression of telomerase enzyme (Duraimani et al., 2015). In another pilot trial, dementia caregivers were exposed to relaxation music, which increased their telomerase activity (Lavretsky et al., 2013). Hence, our study indicates that the maintenance of longer telomeres with the help of Heartfulness meditation is possible.

Our study also focused on the correlation between psychological stress and cellular aging. We examined that non-meditator volunteers who were under stress had shorter telomeres than meditators who showed an increase in telomere length and reduction in psychological and perceived stress. Telomere shortening is a natural process; indicating cellular ageing. But this process is seen to be faster in individuals associated with psychosocial adversity (Thimmapuram et al., 2017). Defects in the DNA repair system can also lead to pathological aging (Pan et al., 2016). Our results show that telomere length is negatively linked to perceived stress and anxiety. The same has been demonstrated in other studies-for anxiety (Hoen et al., 2013) and perceived stress (Epel et al., 2004; Epel, 2009; Epel et al., 2010; Tomiyama et al., 2012; Shalev et al., 2013).

A possible connection between Heartfulness meditation and telomere length may be that individuals who practice Heartfulness experience less stress, anxiety, and depression, which leads to decrease in cortisol levels and this decrease may be associated with enhanced telomerase activity (Schutte and Malouff, 2014). Evidences show that stress-related health problems can be due to cellular ageing, resulting in shortening of telomere length (Epel et al., 2004; Shalev, 2012; Conklin et al., 2018). Therefore, it is important to maintain telomere length for a better cellular health. Healthy life style interventions can possibly increase telomere length in these individuals (Puterman et al., 2018). Findings from Marta et al. show that experienced Zen meditators had longer telomeres as compared to non-meditators (Alda et al., 2016). Similarly, Loving-Kindness meditation practice; another type of meditation showed longer relative telomere length than the control (Hoge et al., 2013).

Researchers have increasingly attributed shorter telomere length to psychosocial stress (Epel et al., 2004; O’Donovan et al., 2011; Drury et al., 2012; Puterman et al., 2016). This study also showed a negative correlation between telomere length, perceived stress and cortisol (as shown in Figures 3A,E). This indicates that the decrease in cortisol (stress) might be due to the increase in telomere length. A recent systematic review and meta-analysis found the same correlation; however, it was between cortisol reactivity in saliva to psychosocial stressors and telomere length and not basal serum cortisol levels in serum and telomere length, as seen in our study (Jiang et al., 2019). The possible mechanism behind improving mental health through Heartfulness intervention could be that the yogic transmission might have led to a state of Samadhi among the participants, leading to an overall positive impact. This involves reduction in perceived and psychological stress leading to changes in the brain via hypothalamic–pituitary–adrenal (HPA) axis (Tolahunase et al., 2017). A key component of the HPA axis is the interaction between the hypothalamus, the pituitary gland, and the adrenal glands, which secrete cortisol; an end result of feedback interactions amongst these glands (Jiang et al., 2019). The reduction in cortisol levels post intervention may have led to an increase in telomere length, which can further slowdown the process of cellular ageing. Researchers have linked this activity in the HPA axis to telomere length (Tomiyama et al., 2012; Savolainen et al., 2015; Nelson et al., 2018). Our findings further show a positive link between well-being and telomere length, hence indicating improved cellular health. These changes in cortisol and telomere length after Heartfulness intervention suggests that Heartfulness can affect our body at cellular and genetic levels and also may slowdown the progression of diseases related to cellular aging.

## Limitations and future research

This study has established a relationship between Heartfulness meditation with modern scientific discipline and provided scientific validation for improving overall well-being by using a non-invasive lifestyle intervention like Heartfulness meditation. However, our study has certain limitations. First, certain parameters did not show a significant effect after the practice of Heartfulness meditation. One reason could be due to the smaller sample size studied in this research. Second, due to the short period of intervention (only 12-weeks of intervention). Third, this study was conducted with only healthy volunteers.

Future studies will include a larger sample size and a longer duration of Heartfulness meditation to strengthen our findings. Additionally, being a part of medical college and a 1,000 bedded hospital, we would like to extend this study to patients with non-communicable lifestyle disorders such as cardiovascular disorder, diabetes, etc. We would also like to study the immunomodulatory patterns resulting due to heartfulness meditation so that we can develop a process and protocol to help quantitative measurement of biological markers. In future, this intervention protocol can be used as a cost-effective and sustainable secondary prevention strategy to maintain well-being.

## Conclusion

The practice of Heartfulness meditation had a positive effect on anxiety, perceived stress, mindfulness, and well-being. The biological indicators such as cortisol concentration and telomere length were also altered after the intervention; showing a reduction in the cortisol levels and an increase in the telomere length. This further indicates that long-term practice of Heartfulness meditation can further improve mental health along with a slow cellular aging process, and hence promote good well-being.

## Data availability statement

The datasets presented in this article are not readily available because informed consent signed by participants stated that data were only accessible to the authors of this study. Requests to access the datasets should be directed to MT, mansibiotech79@gmail.com.

## Ethics statement

The studies involving human participants were reviewed and approved by Ethics Review Committee, of MGMIHS (MGM/DCH/IEC/109/22). The patients/participants provided their written informed consent to participate in this study.

## Author contributions

MT and JT designed the study. YP, SP, and TH conducted the study. SP and TH collected the research data. MT, YP, and KT supervised this work. Results interpretation was done by MT, YP, JT, and NV. SP performed the statistical analyses. SP and MT wrote the original manuscript. JT, NV, and KT reviewed the original manuscript. All authors contributed to the article and approved the submitted version.

## Conflict of interest

NV is employed by Logical Life Science, Pvt. Ltd., Pune, India.

The remaining authors declare that the research was conducted in the absence of any commercial or financial relationships that could be construed as a potential conflict of interest.

## Publisher’s note

All claims expressed in this article are solely those of the authors and do not necessarily represent those of their affiliated organizations, or those of the publisher, the editors and the reviewers. Any product that may be evaluated in this article, or claim that may be made by its manufacturer, is not guaranteed or endorsed by the publisher.

## References

[ref1] Alda M. Puebla-Guedea M. Rodero B. Demarzo M. Montero-Marin J. Roca M.. (2016). Zen meditation, length of telomeres, and the role of experiential avoidance and compassion. Mindfulness 7, 651–659. doi: 10.1007/s12671-016-0500-5, PMID: 27217844 PMC4859856

[ref2] Álvarez-López M. J. Conklin Q. A. Cosín-Tomás M. Shields G. S. King B. G. Zanesco A. P.. (2022). Changes in the expression of inflammatory and epigenetic-modulatory genes after an intensive meditation retreat. Compr. Psychoneuroendocrinol 11:100152. doi: 10.1016/j.cpnec.2022.100152, PMID: 35818436 PMC9270205

[ref3] An A. Hoang H. Trang L. Vo Q. Tran L. Le T.. (2022). Investigating the effect of mindfulness-based stress reduction on stress level and brain activity of college students. IBRO Neurosci. Rep. 12, 399–410. doi: 10.1016/j.ibneur.2022.05.004, PMID: 35601693 PMC9121238

[ref4] Baer R. A. Smith G. T. Lykins E. Button D. Krietemeyer J. Sauer S.. (2008). Construct validity of the five facet mindfulness questionnaire in meditating and nonmeditating samples. Assessment 15, 329–342. doi: 10.1177/1073191107313003, PMID: 18310597

[ref5] Bédard M. Felteau M. Marshall S. Cullen N. Gibbons C. Dubois S.. (2014). Mindfulness-based cognitive therapy reduces symptoms of depression in people with a traumatic brain injury: results from a randomized controlled trial. J. Head Trauma Rehabil. 29, E13–E22. doi: 10.1097/HTR.0b013e3182a615a0, PMID: 24052092

[ref6] Beery A. K. Lin J. Biddle J. S. Francis D. D. Blackburn E. H. Epel E. S. (2012). Chronic stress elevates telomerase activity in rats. Biol. Lett. 8, 1063–1066. doi: 10.1098/rsbl.2012.0747, PMID: 23054915 PMC3497144

[ref7] Blackburn E. H. (2000). Telomere states and cell fates. Nature 408, 53–56. doi: 10.1038/35040500, PMID: 11081503

[ref8] Blackburn E. H. (2009). Telomeres and telomerase: the means to the end. Angew. Chem. Int. Ed. Engl. 49, 7405–7421. doi: 10.1002/anie.201002387 20821774

[ref9] Boccardi V. Paolisso G. Mecocci P. (2016). Nutrition and lifestyle in healthy aging: the telomerase challenge. Aging (Albany NY) 8, 12–15. doi: 10.18632/aging.100886, PMID: 26826704 PMC4761710

[ref10] Bottaccioli A. G. Bottaccioli F. Carosella A. Cofini V. Muzi P. Bologna M. (2020). Psychoneuroendocrinoimmunology-based meditation (PNEIMED) training reduces salivary cortisol under basal and stressful conditions in healthy university students: results of a randomized controlled study. Explore 16, 189–198. doi: 10.1016/j.explore.2019.10.006, PMID: 31982328

[ref11] Cawthon R. M. (2002). Telomere measurement by quantitative PCR. Nucleic Acids Res. 30, e47–e447. doi: 10.1093/nar/30.10.e47, PMID: 12000852 PMC115301

[ref12] Conklin Q. A. King B. G. Zanesco A. P. Lin J. Hamidi A. B. Pokorny J. J.. (2018). Insight meditation and telomere biology: the effects of intensive retreat and the moderating role of personality. Brain Behav. Immun. 70, 233–245. doi: 10.1016/j.bbi.2018.03.003, PMID: 29518528

[ref13] Cramer H. Anheyer D. Lauche R. Dobos G. (2017). A systematic review of yoga for major depressive disorder. J. Affect. Disord. 213, 70–77. doi: 10.1016/j.jad.2017.02.006, PMID: 28192737

[ref14] Cramer H. Lauche R. Langhorst J. Dobos G. (2013). Yoga for depression: a systematic review and meta-analysis. Depress. Anxiety 30, 1068–1083. doi: 10.1002/da.22166 23922209

[ref15] Dada T. Mittal D. Mohanty K. Faiq M. A. Bhat M. A. Yadav R. K.. (2018). Mindfulness meditation reduces intraocular pressure, lowers stress biomarkers and modulates gene expression in glaucoma: a randomized controlled trial. J. Glaucoma 27, 1061–1067. doi: 10.1097/IJG.0000000000001088, PMID: 30256277

[ref16] Davis K. Goodman S. H. Leiferman J. Taylor M. Dimidjian S. (2015). A randomized controlled trial of yoga for pregnant women with symptoms of depression and anxiety. Complement. Ther. Clin. Pract. 21, 166–172. doi: 10.1016/j.ctcp.2015.06.005, PMID: 26256135

[ref17] De Manincor M. Bensoussan A. Smith C. A. Barr K. Schweickle M. Donoghoe L. L.. (2016). Individualized yoga for reducing depression and anxiety, and improving well-being: a randomized controlled trial. Depress. Anxiety 33, 816–828. doi: 10.1002/da.22502 27030303

[ref18] Drury S. S. Theall K. Gleason M. M. Smyke A. T. De Vivo I. Wong J.. (2012). Telomere length and early severe social deprivation: linking early adversity and cellular aging. Mol. Psychiatry 17, 719–727. doi: 10.1038/mp.2011.53, PMID: 21577215 PMC3518061

[ref19] Duraimani S. Schneider R. H. Randall O. S. Nidich S. I. Xu S. Ketete M.. (2015). Effects of lifestyle modification on telomerase gene expression in hypertensive patients: a pilot trial of stress reduction and health education programs in African Americans. PLoS One 10:e0142689. doi: 10.1371/journal.pone.0142689, PMID: 26571023 PMC4646647

[ref20] Dyrbye L. N. Thomas M. R. Massie F. S. Power D. V. Eacker A. Harper W.. (2008). Burnout and suicidal ideation among US medical students. Ann. Intern. Med. 149, 334–341. doi: 10.7326/0003-4819-149-5-200809020-00008, PMID: 18765703

[ref21] Epel E. S. (2009). Telomeres in a life-span perspective: a new “psychobiomarker”? Curr. Dir. Psychol. Sci. 18, 6–10. doi: 10.1111/j.1467-8721.2009.01596

[ref22] Epel E. S. Blackburn E. H. Lin J. Dhabhar F. S. Adler N. E. Morrow J. D.. (2004). Accelerated telomere shortening in response to life stress. Proc. Natl. Acad. Sci. U. S. A. 101, 17312–17315. doi: 10.1073/pnas.0407162101, PMID: 15574496 PMC534658

[ref23] Epel E. S. Lin J. Dhabhar F. S. Wolkowitz O. M. Puterman E. Karan L.. (2010). Dynamics of telomerase activity in response to acute psychological stress. Brain Behav. Immun. 24, 531–539. doi: 10.1016/j.bbi.2009.11.018, PMID: 20018236 PMC2856774

[ref24] Falsafi N. (2016). A randomized controlled trial of mindfulness versus yoga: effects on depression and/or anxiety in college students. J. Am. Psychiatr. Nurses Assoc. 22, 483–497. doi: 10.1177/1078390316663307 27566622

[ref25] Faul F. Erdfelder E. Lang A. G. Buchner A. (2007). G* Power 3: a flexible statistical power analysis program for the social, behavioral, and biomedical sciences. Behav. Res. Methods 39, 175–191. doi: 10.3758/BF03193146, PMID: 17695343

[ref26] Gardi C. Fazia T. Stringa B. Giommi F. (2022). A short mindfulness retreat can improve biological markers of stress and inflammation. Psychoneuroendocrinology 135:105579. doi: 10.1016/j.psyneuen.2021.105579, PMID: 34775250

[ref27] Goldberg S. B. Tucker R. P. Greene P. A. Davidson R. J. Wampold B. E. Kearney D. J.. (2018). Mindfulness-based interventions for psychiatric disorders: a systematic review and meta-analysis. Clin. Psychol. Rev. 59, 52–60. doi: 10.1016/j.cpr.2017.10.011, PMID: 29126747 PMC5741505

[ref28] Haycock P. C. Heydon E. E. Kaptoge S. Butterworth A. S. Thompson A. Willeit P. (2014). Leucocyte telomere length and risk of cardiovascular disease: systematic review and meta-analysis. BMJ 349:g4227. doi: 10.1136/bmj.g4227, PMID: 25006006 PMC4086028

[ref29] Hilcove K. Marceau C. Thekdi P. Larkey L. Brewer M. A. Jones K. (2021). Holistic nursing in practice: mindfulness-based yoga as an intervention to manage stress and burnout. J. Holist. Nurs. 39, 29–42. doi: 10.1177/0898010120921587, PMID: 32460584

[ref30] Hilton L. Maher A. R. Colaiaco B. Apaydin E. Sorbero M. E. Booth M.. (2017). Meditation for posttraumatic stress: systematic review and meta-analysis. Psychol. Trauma Theory Res. Pract. Policy 9, 453–460. doi: 10.1037/tra0000180, PMID: 27537781

[ref31] Hoen P. W. Rosmalen J. G. M. Schoevers R. A. Huzen J. Van Der Harst P. De Jonge P. (2013). Association between anxiety but not depressive disorders and leukocyte telomere length after 2 years of follow-up in a population-based sample. Psychol. Med. 43, 689–697. doi: 10.1017/S0033291712001766 22877856

[ref32] Hofmann S. G. Sawyer A. T. Witt A. A. Oh D. (2010). The effect of mindfulness-based therapy on anxiety and depression: a meta-analytic review. J. Consult. Clin. Psychol. 78, 169–183. doi: 10.1037/a0018555, PMID: 20350028 PMC2848393

[ref33] Hoge E. A. Bui E. Palitz S. A. Schwarz N. R. Owens M. E. Johnston J. M.. (2018). The effect of mindfulness meditation training on biological acute stress responses in generalized anxiety disorder. Psychiatry Res. 262, 328–332. doi: 10.1016/j.psychres.2017.01.006, PMID: 28131433 PMC5526744

[ref34] Hoge E. A. Chen M. M. Orr E. Metcalf C. A. Fischer L. E. Pollack M. H.. (2013). Loving-kindness meditation practice associated with longer telomeres in women. Brain Behav. Immun. 32, 159–163. doi: 10.1016/j.bbi.2013.04.005, PMID: 23602876

[ref35] Hwang E. Y. Chung S. Y. Cho J. H. Song M. Y. Kim S. Kim J. W. (2013). Effects of a brief qigong-based stress reduction program (BQSRP) in a distressed Korean population: a randomized trial. BMC Complement. Altern. Med. 13, 1–7. doi: 10.1186/1472-6882-13-113 23705963 PMC3680074

[ref36] Jacobs T. L. Epel E. S. Lin J. Blackburn E. H. Wolkowitz O. M. Bridwell D. A.. (2011). Intensive meditation training, immune cell telomerase activity, and psychological mediators. Psychoneuroendocrinology 36, 664–681. doi: 10.1016/j.psyneuen.2010.09.010, PMID: 21035949

[ref37] Jiang Y. Da W. Qiao S. Zhang Q. Li X. Ivey G.. (2019). Basal cortisol, cortisol reactivity, and telomere length: a systematic review and meta-analysis. Psychoneuroendocrinology 103, 163–172. doi: 10.1016/j.psyneuen.2019.01.022, PMID: 30695740 PMC6450740

[ref38] Karthik L. Kumar G. Keswani T. Bhattacharyya A. Chandar S. S. Bhaskara Rao K. V. (2014). Protease inhibitors from marine actinobacteria as a potential source for antimalarial compound. PLoS One 9:e90972. doi: 10.1371/journal.pone.0090972, PMID: 24618707 PMC3949715

[ref39] Kiran U. Ladha S. Makhija N. Kapoor P. M. Choudhury M. Das S.. (2017). The role of Rajyoga meditation for modulation of anxiety and serum cortisol in patients undergoing coronary artery bypass surgery: a prospective randomized control study. Ann. Card. Anaesth. 20, 158–162. doi: 10.4103/aca.ACA_32_17, PMID: 28393774 PMC5408519

[ref40] Lavretsky H. Epel E. S. Siddarth P. Nazarian N. Cyr N. S. Khalsa D. S.. (2013). A pilot study of yogic meditation for family dementia caregivers with depressive symptoms: effects on mental health, cognition, and telomerase activity. Int. J. Geriatr. Psychiatry 28, 57–65. doi: 10.1002/gps.3790, PMID: 22407663 PMC3423469

[ref41] Lee E. H. (2012). Review of the psychometric evidence of the perceived stress scale. Asian Nurs. Res. (Korean Soc. Nurs. Sci.) 6, 121–127. doi: 10.1016/j.anr.2012.08.004, PMID: 25031113

[ref42] Lengacher C. A. Reich R. R. Kip K. E. Barta M. Ramesar S. Paterson C. L.. (2014). Influence of mindfulness-based stress reduction (MBSR) on telomerase activity in women with breast cancer (BC). Biol. Res. Nurs. 16, 438–447. doi: 10.1177/1099800413519495, PMID: 24486564 PMC4559344

[ref43] Lipton B. H., (2008). The Biology of Belief–Unleashing the Power of Consciousness Matter & Miracles, Hay House. Carlsbad, CA.

[ref44] Michalsen A. Jeitler M. Brunnhuber S. Lüdtke R. Büssing A. Musial F.. (2012). Iyengar yoga for distressed women: a 3-armed randomized controlled trial. eCAM 2012, 1–9. doi: 10.1155/2012/408727, PMID: 23049608 PMC3463199

[ref45] Nelson B. W. Allen N. B. Laurent H. (2018). Infant HPA axis as a potential mechanism linking maternal mental health and infant telomere length. Psychoneuroendocrinology 88, 38–46. doi: 10.1016/j.psyneuen.2017.11.008, PMID: 29161636

[ref46] Nijjar P. S. Connett J. E. Lindquist R. Brown R. Burt M. Pergolski A.. (2019). Randomized trial of mindfulness-based stress reduction in cardiac patients eligible for cardiac rehabilitation. Sci. Rep. 9, 1–11. doi: 10.1038/s41598-019-54932-2 31804580 PMC6895078

[ref47] O’Donovan A. Epel E. Lin J. Wolkowitz O. Cohen B. Maguen S.. (2011). Childhood trauma associated with short leukocyte telomere length in posttraumatic stress disorder. Biol. Psychiatry 70, 465–471. doi: 10.1016/j.biopsych.2011.01.035, PMID: 21489410 PMC3152637

[ref48] Pace T. W. Negi L. T. Adame D. D. Cole S. P. Sivilli T. I. Brown T. D.. (2009). Effect of compassion meditation on neuroendocrine, innate immune and behavioral responses to psychosocial stress. Psychoneuroendocrinology 34, 87–98. doi: 10.1016/j.psyneuen.2008.08.011, PMID: 18835662 PMC2695992

[ref49] Pan M. R. Li K. Lin S. Y. Hung W. C. (2016). Connecting the dots: from DNA damage and repair to aging. Int. J. Mol. Sci. 17:685. doi: 10.3390/ijms17050685, PMID: 27164092 PMC4881511

[ref50] Parthasarathy S. Jaiganesh K. (2014). Effect of integrated yoga module on selected psychological variables among women with anxiety problem. West Indian Med. J. 63, 78–80. doi: 10.7727/wimj.2012.054, PMID: 25303199 PMC4655636

[ref51] Piotrowski C. (1999). The status of the Beck anxiety inventory in contemporary research. Psychol. Rep. 85, 261–262. doi: 10.2466/pr0.1999.85.1.261, PMID: 10575991

[ref52] Prathikanti S. Rivera R. Cochran A. Tungol J. G. Fayazmanesh N. Weinmann E. (2017). Treating major depression with yoga: a prospective, randomized, controlled pilot trial. PLoS One 12:e0173869. doi: 10.1371/journal.pone.0173869, PMID: 28301561 PMC5354384

[ref53] Puterman E. Gemmill A. Karasek D. Weir D. Adler N. E. Prather A. A.. (2016). Lifespan adversity and later adulthood telomere length in the nationally representative US health and retirement study. Proc. Natl. Acad. Sci. 113, E6335–E6342. doi: 10.1073/pnas.1525602113 27698131 PMC5081642

[ref54] Puterman E. Weiss J. Lin J. Schilf S. Slusher A. L. Johansen K. L.. (2018). Aerobic exercise lengthens telomeres and reduces stress in family caregivers: a randomized controlled trial-Curt Richter award paper 2018. Psychoneuroendocrinology 98, 245–252. doi: 10.1016/j.psyneuen.2018.08.002 30266522

[ref55] Ramanathan M. Bhavanani A. B. Trakroo M. (2017). Effect of a 12-week yoga therapy program on mental health status in elderly women inmates of a hospice. Int. J. Yoga 10, 24–28. doi: 10.4103/0973-6131.186156, PMID: 28149064 PMC5225740

[ref56] Ridout K. K. Ridout S. J. Price L. H. Sen S. Tyrka A. R. (2016). Depression and telomere length: a meta-analysis. J. Affect. Disord. 191, 237–247. doi: 10.1016/j.jad.2015.11.052, PMID: 26688493 PMC4760624

[ref57] Savolainen K. Eriksson J. G. Kajantie E. Lahti J. Raikkonen K. (2015). Telomere length and hypothalamic-pituitary-adrenal axis response to stress in elderly adults. Psychoneuroendocrinology 53, 179–184. doi: 10.1016/j.psyneuen.2014.12.020, PMID: 25622010

[ref58] Schutte N. S. Malouff J. M. (2014). A meta-analytic review of the effects of mindfulness meditation on telomerase activity. Psychoneuroendocrinology 42, 45–48. doi: 10.1016/j.psyneuen.2013.12.017, PMID: 24636500

[ref59] Seppälä E. M. Nitschke J. B. Tudorascu D. L. Hayes A. Goldstein M. R. Nguyen D. T.. (2014). Breathing-based meditation decreases posttraumatic stress disorder symptoms in US military veterans: a randomized controlled longitudinal study. J. Trauma. Stress. 27, 397–405. doi: 10.1002/jts.21936, PMID: 25158633 PMC4309518

[ref60] Shalev I. (2012). Early life stress and telomere length: investigating the connection and possible mechanisms: a critical survey of the evidence base, research methodology and basic biology. BioEssays 34, 943–952. doi: 10.1002/bies.201200084, PMID: 22991129 PMC3557830

[ref61] Shalev I. Entringer S. Wadhwa P. D. Wolkowitz O. M. Puterman E. Lin J.. (2013). Stress and telomere biology: a lifespan perspective. Psychoneuroendocrinology 38, 1835–1842. doi: 10.1016/j.psyneuen.2013.03.010, PMID: 23639252 PMC3735679

[ref62] Song Q. H. Shen G. Q. Xu R. M. Zhang Q. H. Ma M. Guo Y. H.. (2014). Effect of tai chi exercise on the physical and mental health of the elder patients suffered from anxiety disorder. Int. J. Physiol. Pathophysiol. Pharmacol. 6, 55–60. PMID: 24665359 PMC3961102

[ref63] Streeter C. C. Gerbarg P. L. Whitfield T. H. Owen L. Johnston J. Silveri M. M.. (2017). Treatment of major depressive disorder with Iyengar yoga and coherent breathing: a randomized controlled dosing study. J. Altern. Complement. Med. 23, 201–207. doi: 10.1089/acm.2016.0140, PMID: 28296480 PMC5359682

[ref64] Sudsuang R. Chentanez V. Veluvan K. (1991). Effect of Buddhist meditation on serum cortisol and total protein levels, blood pressure, pulse rate, lung volume and reaction time. Physiol. Behav. 50, 543–548. doi: 10.1016/0031-9384(91)90543-W, PMID: 1801007

[ref65] Sylapan B. S. Nair A. K. Jayanna K. Mallipeddi S. Sathyanarayana S. Kutty B. M. (2020). Meditation, well-being and cognition in heartfulness meditators–a pilot study. Conscious. Cogn. 86:103032. doi: 10.1016/j.concog.2020.103032, PMID: 33096504

[ref66] Thimmapuram J. Pargament R. Sibliss K. Grim R. Risques R. Toorens E. (2017). Effect of heartfulness meditation on burnout, emotional wellness, and telomere length in health care professionals. J. Community Hosp. Intern. Med. Perspect. 7, 21–27. doi: 10.1080/20009666.2016.1270806, PMID: 28634520 PMC5463663

[ref67] Tolahunase M. Sagar R. Dada R. (2017). Impact of yoga and meditation on cellular aging in apparently healthy individuals: a prospective, open-label single-arm exploratory study. Oxidative Med. Cell. Longev. 2017, 1–9. doi: 10.1155/2017/7928981 PMC5278216 28191278

[ref68] Tolahunase M. R. Sagar R. Faiq M. Dada R. (2018). Yoga-and meditation-based lifestyle intervention increases neuroplasticity and reduces severity of major depressive disorder: a randomized controlled trial. Restor. Neurol. Neurosci. 36, 423–442. doi: 10.3233/RNN-170810, PMID: 29614706

[ref69] Tomiyama A. J. O’Donovan A. Lin J. Puterman E. Lazaro A. Chan J.. (2012). Does cellular aging relate to patterns of allostasis? An examination of basal and stress reactive HPA axis activity and telomere length. Physiol. Behav. 106, 40–45. doi: 10.1016/j.physbeh.2011.11.016, PMID: 22138440 PMC3361080

[ref70] Topp C. W. Østergaard S. D. Søndergaard S. Bech P. (2015). The WHO-5 well-being index: a systematic review of the literature. Psychother. Psychosom. 84, 167–176. doi: 10.1159/000376585, PMID: 25831962

[ref71] Tsang H. W. Tsang W. W. Jones A. Y. Fung K. M. Chan A. H. Chan E. P.. (2013). Psycho-physical and neurophysiological effects of qigong on depressed elders with chronic illness. Aging Ment. Health 17, 336–348. doi: 10.1080/13607863.2012.732035, PMID: 23072658

[ref72] Turan B. Foltz C. Cavanagh J. F. Wallace B. A. Cullen M. Rosenberg E. L.. (2015). Anticipatory sensitization to repeated stressors: the role of initial cortisol reactivity and meditation/emotion skills training. Psychoneuroendocrinology 52, 229–238. doi: 10.1016/j.psyneuen.2014.11.014, PMID: 25497480

[ref73] van’t Westeinde A. Patel K. D. (2022). Heartfulness meditation: a yogic and neuroscientific perspective. Front. Psychol. 13:806131. doi: 10.3389/fpsyg.2022.806131, PMID: 35619781 PMC9128627

[ref74] Vorkapic C. F. Rangé B. (2014). Reducing the symptomatology of panic disorder: the effects of a yoga program alone and in combination with cognitive-behavioral therapy. Front. Psych. 5:177. doi: 10.3389/fpsyt.2014.00177, PMID: 25538634 PMC4259001

[ref75] Walsh R. Shapiro S. L. (2006). The meeting of meditative disciplines and Western psychology: a mutually enriching dialogue. Am. Psychol. 61, 227–239. doi: 10.1037/0003-066X.61.3.227, PMID: 16594839

[ref76] Walvekar S. S. Ambekar J. G. Devaranavadagi B. B. (2015). Study on serum cortisol and perceived stress scale in the police constables. J. Clin. Diagn. Res. 9:BC10. doi: 10.7860/JCDR/2015/12015.5576 25859444 PMC4378726

[ref77] Wang C. Bannuru R. Ramel J. Kupelnick B. Scott T. Schmid C. H. (2010). Tai chi on psychological well-being: systematic review and meta-analysis. BMC Complement. Altern. Med. 10, 1–16. doi: 10.1186/1472-6882-10-23 20492638 PMC2893078

[ref78] Wolkowitz O. M. Mellon S. H. Epel E. S. Lin J. Dhabhar F. S. Su Y.. (2011). Leukocyte telomere length in major depression: correlations with chronicity, inflammation and oxidative stress-preliminary findings. PLoS One 6:e17837. doi: 10.1371/journal.pone.0017837, PMID: 21448457 PMC3063175

[ref79] Yeung A. S. Feng R. Kim D. J. H. Wayne P. M. Yeh G. Y. Baer L.. (2017). A pilot, randomized controlled study of tai chi with passive and active controls in the treatment of depressed Chinese Americans. J. Clin. Psychiatry 78, e522–e528. doi: 10.4088/JCP.16m10772 28570792

[ref80] Yin J. Dishman R. K. (2014). The effect of tai chi and Qigong practice on depression and anxiety symptoms: a systematic review and meta-regression analysis of randomized controlled trials. Ment. Health Phys. Act. 7, 135–146. doi: 10.1016/j.mhpa.2014.08.001

[ref81] Zhao J. Miao K. Wang H. Ding H. Wang D. W. (2013). Association between telomere length and type 2 diabetes mellitus: a meta-analysis. PLoS One 8:e79993. doi: 10.1371/journal.pone.0079993, PMID: 24278229 PMC3836967

[ref82] Zi W. J. Shuai J. (2013). Cortisol as a prognostic marker of short-term outcome in chinese patients with acute ischemic stroke. PLoS One 8:e72758. doi: 10.1371/journal.pone.0072758, PMID: 24069157 PMC3771965

